# Rightful place of qualitative research in family medicine and healthcare

**DOI:** 10.51866/cm0004

**Published:** 2022-11-27

**Authors:** Seng Fah Tong, Chai-Eng Tan

**Affiliations:** 1 MD (UKM), MMed (Fam Med) (UKM), Department of Family Medicine, Faculty of Medicine, Universiti, Kebangsaan Malaysia, Jalan, Yaacob Latif, Cheras, Kuala Lumpur, Malaysia. Email: tce@ppukm.ukm.edu.my; 2 MBBS (UM), MMed(FamMed)(UKM), PhD (Sydney), Department of Family Medicine, Faculty of Medicine, Universiti, Kebangsaan Malaysia, Jalanm Yaacob Latif, Cheras, Kuala Lumpur, Malaysia.

**Keywords:** Family practice, Evidence-based medicine, Qualitative research

## Abstract

Evidence-based medicine is the foundation of current medical practice. Suitable evidence is needed to support the holistic approach in clinical practice. Quantitative research produces some evidence needed for disease treatment based on probabilities or averages. However, the practice of evidence-based medicine should be personalised to individual patients without relying solely on an average perspective. Beliefs, values and expectations are unique for each individual and may differ significantly from the average. Therefore, understanding individual differences requires evidence from qualitative research. This is particularly important in family medicine practice, which focuses on holistic care for the person, family and community. Findings from properly conducted qualitative research can offer in-depth and comprehensive accounts on healthcare issues from patient and practice perspectives. Qualitative research also provides explanatory power and analytical transferability, which can be applied into daily family medicine practice. In conclusion, evidence from qualitative research should be rightfully equally acknowledged in family medicine and healthcare.

## Introduction

Evidence-based medicine is the foundation of current medical practice. Despite advancement in research to generate new evidence, disease management in clinical practice remains challenging, especially for chronic diseases. The majority of evidence arises from quantitative research, which has provided information on optimal treatment of diseases. In particular, quantitative research has provided knowledge regarding treatment targets for many diseases and their treatment options. However, optimal control of diseases remains difficult in some patients. Multidisciplinary care is essential in disease management; yet, establishing an efficient and effective team remains challenging. Although the necessary measures are known, knowledge of how to effectively implement them is limited.

Such challenges have common issues, whereby human and social factors are major determinants of their success. Understanding human behaviour and social environment then becomes important for interventions to be impactful. Therefore, research evidence that can help in understanding human behaviour and social environment better is necessary to support clinical practice. Quantitative research has limitations in this aspect because human behaviour and social interactions are difficult to measure. Qualitative research is more suited to address this need.

Professions that relate closely with human behaviour acknowledge the role of qualitative research.^1^ The rightful role of evidence from qualitative research in family medicine and healthcare must be recognised. In this article, we will briefly describe the nature of family medicine practice, discuss how family medicine intersects with qualitative research and position the usefulness of qualitative evidence in family medicine and healthcare in general.

## Definition of family medicine and the needed evidence to support its practice

In contrast to other specialties that focus on specific physiological systems, family medicine is not easily defined. While this article does not intend to argue for a particular definition, family medicine can be seen as a specialty that focuses on people.^2^ This means that family medicine focuses on the patient as a whole person and as an individual, as opposed to public health, which focuses on the community. Medical decisions in family medicine are made after taking into account the bio-psychosocial and spiritual well-being of patients. Shared decision-making between physicians and patients is an integral part of this process. Active participation of patients in the form of adjusting their behaviours and daily routines is needed to accommodate the treatment plan agreed on, especially in managing chronic illnesses. This is consistent with the concept of evidence-based practice proposed originally by Sackett et al.^3,4^ Patients’ ideas, beliefs, perceptions and expectations must also be evaluated in relation to their social well-being and relationship before a particular treatment plan is made.^4^ Managing patients’ psychological and social well-being is equally important as treating diseases.

Individuals construct their own meanings of diseases and illnesses and their expectations to treatment. Often, these meanings are built from their life and lived experiences as well as the surrounding social structure and dynamics, which vary among patients. For example, one patient may view diabetes as a common illness that is inherited from parents, whereas another patient may view it as a serious consequence of his/her own lifestyle. Hence, these two patients may react to the need to start medications or institute a lifestyle change differently. There are numerous sociocultural influences on patients’ behavioural and social adjustments, such as family and community values, beliefs and cultural norms. Understanding and acknowledging these can facilitate communication with empathy, to assess patients’ concerns more accurately and to make better decisions in personalising their treatment plans.

An effective and efficient family medicine practice cannot function in isolation. Family medicine clinics often include a team of different healthcare professions, such as nurses, pharmacists, dietitians and rehabilitation therapists. The effectiveness of family medicine practice also depends on the teamwork and interprofessional collaborations between these healthcare professionals. Teamwork and healthcare processes can potentially influence the success of patient management.

Given this perspective of family medicine, two types of evidence are necessary. The first type provides the probability of treatment effectiveness (what commonly works for a disorder) or how impactful an issue is (the magnitude of an issue). This type of evidence can help in deciding what will probably work for a disorder and what should be prioritised in healthcare management.^5^ The second type provides an understanding of a patient as a holistic individual.^5^

The first type of evidence uses the quantitative approach to provide a probability perspective. It answers the following questions: What is the usual probability of a treatment that will work for a typical patient? For example, what is the usual odds of metformin in reducing the mortality rate of a typical adult Asian patient with type 2 diabetes? What is the usual knowledge level about diabetes and its association with diabetes control? Statistics generated in the quantitative approach are centred around a point estimate from a sampled population, such as a mean or a median. This point estimate does not reflect any particular individual but provides a representation of the ‘average’ population. Further, during patient treatment, there is no ‘average’ because only a single individual is being managed. It either works or fails for that particular individual. With evidence from quantitative data, the probability of effectiveness of the treatment based on the knowledge of the ‘average’ can be estimated. However, the actual benefits obtained depend on the individual context.

This leads to the role of the second type of evidence, which can help in understanding a patient as a complete person. The qualitative approach is needed to understand the possible spectrum and type of logic that may fit each patient. An approach that allows measurable and unmeasurable variables to be examined together is needed to examine and understand the complexity of family medicine practice. These pieces of evidence are often interconnected and should not be examined in isolation. This can potentially be accomplished with the qualitative approach.

## Qualitative research relevant to family medicine practice

Qualitative research has many definitions depending on the perspective. In the selection of a methodology based on research questions, qualitative research can be defined as a scientific investigative methodology suited for research questions aimed at understanding phenomena related primarily to human and social environments. Qualitative research may utilise any type of empirical data with its interpretive methods. It does not evaluate the magnitude of a phenomenon, strength of an association or effect size, in contrast to quantitative research. It focuses on understanding evidence from empirical data by answering the ‘what’, ‘how’ and ‘why’.

Qualitative research is well positioned in medicine and health research because of the fundamental intersection of medicine and health with humanity and social sciences. It is an optimal approach for exploring the layers of human complexity in the practice of family medicine and healthcare. Research questions related to understanding medical humanities may be arbitrarily divided into three categories: i) exploring the spectrum of a phenomenon, ii) exploring patient-constructed meaning and iii) explaining actions, health behaviours or occurrence of a phenomenon. These research questions are best answered using a specific qualitative research design. The general qualitative approach does not fit all types of research questions.

### Exploring the spectrum of a phenomenon

A phenomenon refers to an incidence of interest. The intention of being exploratory is justified when the spectrum is not well known in the literature. Such exploration focuses on the ‘what’, which may include the following: 1) behaviours of interest (e.g. eating patterns of patients with diabetes), 2) factors contributing to a phenomenon (e.g. factors associated with adherence to clinic attendance) or 3) views and perceptions towards a primary care service programme (e.g. perceptions towards a virtual clinic for a chronic disease). The qualitative approach answers the ‘what’ question by generating comprehensive themes. The findings should be comprehensive by covering all possibilities of behavioural patterns, factors and views.

To further illustrate the use of qualitative research in exploring the spectrum of a phenomenon, we will provide some examples. Wong et al. explored the factors that influence the help-seeking behaviour of caregivers to patients with first-episode psychosis.^6^ From their qualitative inquiry, they presented a list of themes with corresponding subthemes. The themes represented the spectrum of internal factors for knowledge and stigma related to schizophrenia. The analysis also revealed a spectrum of four types of help-seeking behaviour based on the two factors identified. Caregivers seek help either early or very late depending on the interplay between the knowledge of schizophrenia and the associated stigma. Pickles et al. investigated doctors’ approaches to prostate-specific antigen (PSA) testing in the primary healthcare setting.^7^ They identified a spectrum of behaviour, which ranged from doctors being highly likely to offer PSA testing, doctors weighing the advantages and disadvantages before offering PSA testing and doctors offering it only upon patients’ request to doctors being reluctant to order PSA testing. The findings can help identify patients or physicians within the spectra presented. No one is considered an outlier.

The exploratory nature of the qualitative approach enables the investigation of spectra that may be unknown or unexpected. This approach does not require pre-determined concepts or their corresponding indicators, which may limit the extent of exploration. The methodology also allows flexibility and creative use of data collection methods. Exploratory qualitative research often uses unstructured or semi-structured interviews, focus group discussions and observations. With the use of a good systematic qualitative methodology to achieve saturation (Box 1), the findings are often comprehensive, providing all possible ‘differentials’ or potential insights on the investigated phenomenon.

Box 1 **Saturation.**

Saturation is the decision made by researchers when further data gathering will neither provide additional understanding nor alter the study findings.^8^ There are three types of saturation:

1. data saturation^*^
2. thematic or analytical saturation
3. theoretical saturation

*Data saturation is the weakest type of saturation.

A qualitative approach does not aim to make a single general statement regarding the findings because the findings focus on the spectrum. For example, the statement ‘We found that the intervention was well accepted by patients’ has a quantitative connotation. It implies the summed perception of patients in the study. In another example, the statement ‘We found that the knowledge of patients about Pap smear is generally poor’ would only be credible if it was based on data gathered using probabilistic sampling from the intended population. Therefore, researchers should be mindful of their choice of words when concluding their findings in qualitative research.

Saturation determines whether the findings of exploratory qualitative research are adequately comprehensive. Often, readers of qualitative research articles can also judge whether the findings have covered all possible spectra. If readers find that ‘there is nothing more to add’, then saturation is likely to have been achieved. When the uncovered spectrum is all encompassing, it justifies the full exploratory intention of the qualitative approach.

### Exploring patient-constructed meaning

The meaning constructed by patients is closely related to their health behaviour. Patient-constructed meaning can refer to their personal beliefs, values and attitudes towards an illness and its treatment. Beliefs are ideas that are perceived as the truth, whereas values are things or ideas that one holds as important. Attitudes are the thoughts of how one would respond to a particular situation. These three concepts are interrelated. Beliefs and values commonly precede attitude. If beliefs and values are shared within a community or social group, they become cultural beliefs and values.

Beliefs, values and general attitudes may not be accurate in predicting a specific behaviour but exert their influence on a wide range of behaviours.^9^ Therefore, it is important to uncover these three concepts because they can help in understanding patients’ ideas, concerns and expectations when discussing treatment strategies. An exploratory design is needed to uncover all possible beliefs and values given their vast range. Because meanings are abstract, an in-depth and detailed account of patients’ feelings or thoughts in their lived experience is required to make sense of the patient-constructed meaning. The qualitative approach is best suited to explore such accounts.

One example of how qualitative research is used to explore patient-constructed meaning is described in the paper by Peterson et al., who investigated the meaning of diabetes among patients.^10^ The authors noted that patients used many methods and underwent different phases from being passive to assuming control in their life living with diabetes.^10^ The meanings changed throughout the patients’ life, and the concept of diabetes transitioned from background to foreground and vice versa every minute of their everyday life.^11^ Youngson et al. described the metaphor of patients living with diabetes being akin to charting a course of health and well-being through a choppy sea.^12^ The boat represented the patients making their journey through a choppy physical and social environment, with the help of healthcare providers at the boatyard. This metaphor helps readers understand that patients with diabetes would need to take control in balancing their life and condition, without a moment of stopping, until they reach the boatyard.^12^ Such in-depth accounts can help in further understanding patients’ perspectives.

The above-indicated examples can aid in empathising with patients during consultations, which can promote deeper understanding. An intervention may seem logical and appropriate from physicians’ perspectives but could be totally irrelevant from patients’ perspectives. This concept is one of the key components in shared decision-making. A good qualitative study aimed at exploring meanings should provide an analysis with sufficient depth, instead of merely describing the findings. Phenomenology is a specific qualitative method that is often adopted for this type of research questions.^13^ Although the analysis is thematic, a properly executed phenomenological study should achieve a level of abstraction that provides sufficient explanatory power. In-depth analysis helps increase the transferability of findings beyond the research setting, including clinical practice.

### Explaining actions, health behaviours or occurrence of a phenomenon

It is essential to identify what determines or predicts an action, so that interventions can be designed to promote desirable health behaviours. Researchers are attempting to formulate a theory or some parts of it to link the determinants and predictors with an action. A theory consists of interrelated groups of concepts that explain or predict an event or outcome.^14^ A theory from a qualitative perspective differs from modelling in statistics, which attempts to construct a model based on statistics to represent a group of a sampled population. As opposed to the population level, health behavioural theories that have explanatory power at the individual level are needed. Determinants of health behaviours are complex with many interconnected variables. For example, a thinking process can lead to behavioural intentions that result in a health behaviour.^15^ This type of theory can arise from qualitative research.

At the organisational level of family medicine practice, theories that can help explain and improve practice are essential.^16,17^ The effectiveness of various interventions based on these theories varies in different settings, and some of them may fail.18 Interventions are at a higher risk of failure if the selected underpinning theory involves only a few concepts without understanding their interaction with other determinants. Interventions based on generic theories that do not fit the context or discipline are also at a higher risk of failure.^18^ Thus, the qualitative approach may be needed to explore concepts relevant to local contexts that could influence potential interventions. This type of research approach focuses on ‘why’ or ‘how’ events happen in the local context.

Cheong et al. explored the factors associated with intention to undertake cardiovascular health checks.^19^ Using the grounded theory approach, they identified a core category (or core concept) — the deciding theme that explained an individual’s subsequent action. The intention to undergo health checks is the sum of the perceived relevance and readiness to face the outcomes of such checks.^19^ During clinic consultations, the importance of health checks must be conveyed to patients, and the readiness to accept the subsequent management plans following the results of these health checks must be addressed.^19^ In their study on general practitioners’ behaviour on PSA testing, Pickles et al. were able to relate the explanations for different types of decision-making to the general practitioners’ past experiences, medicolegal obligations, guidelines and evidence for PSA testing. The interplay between these factors determined which of the four decision-making approaches will be adopted.^7^ From this finding, the authors proposed that future interventions should be more sensitive to general practitioners’ experiences, as merely informing the evidence concerning PSA testing may be insufficient to change their behaviour. These two studies provide examples of qualitative findings presented in a story-telling format with conceptual models.

The role of qualitative research is similar at the organisational level. McVea et al. explained how the ‘Put Prevention into Practice’ programme was successful in some clinics but not in other clinics.^20^ They found that preventive focused organisation and readiness to change were two key components of the success of the health prevention programme in clinics.^20^ This example highlights how qualitative research can generate a theory that will be useful in family medicine practice.

For a theory to be useful in clinical practice, the developed concepts need to be logically connected and comprehensive, covering all significant determinants to the extent possible. A theory needs to be stable and transferable to situations beyond the research context. This requires theoretical saturation, not merely thematic saturation. In theoretical saturation, further data do not add new concepts in the construction of a theory. Thus, the resulting theory would be stable and relevant beyond the research setting and applicable to clinical practice.

Several qualitative approaches may be used to achieve these objectives. The grounded theory approach, which uses systematic steps towards theoretical saturation, is the usual choice for constructing an explanatory theory.^21^ The case study approach may be suitable if the phenomenon of interest is investigated in relation to its bounded socio-geographical context.^22^ In qualitative case studies, in-depth description and cross-case examination are required during analysis to provide a theoretical explanation to the phenomenon in context. Another option is to use an exploratory descriptive thematic design. However, this usually produces a list of themes without connection between the themes. Therefore, this design may lack the explanatory power of how the concepts evolve to produce an outcome.

## Conclusion

Qualitative research offers various means to explore and understand a phenomenon in greater depth as well as to generate a theoretical explanation for such a phenomenon. A rigorously conducted qualitative study with an in-depth analysis can provide explanatory representativeness and analytical transferability beyond the research context.^23^ Qualitative researchers should thus aim to go beyond describing data and to obtain new knowledge through in-depth interpretation and analysis. Only then can qualitative research generate relevant knowledge that is valuable for family medicine clinicians, improving empathy and understanding towards patients and guiding clinical decision-making and design of individual- and practice-level interventions. Theories generated from qualitative research can potentially form valuable frameworks for future research. In conclusion, qualitative research has an important role in primary care: It offers much needed evidence for problem-solving in primary care and complements evidence from quantitative research.
